# Supplementary material for: Antimicrobial Resistance and Prevalence of Extended Spectrum β-Lactamase-Producing Escherichia coli from Dogs and Cats in Northeastern China from 2012 to 2021
Source: Antibiotics (Basel). 2022 Oct 28;11(11):1506. doi: 10.3390/antibiotics11111506 (PMC9686880; doi:10.3390/antibiotics11111506)
Supplement: Supplementary file 1 [file antibiotics-11-01506-s001.zip › antibiotics-1923183-supplementary.pdf]

antibiotics MDPI

Supplementary File

Table S1. Antibacterial resistance patterns for *E. coli*.

Antibiotic resistance patterns	Amount
AMK	6
AMP	3
ATM	1
GEN	1
SXT	1
TET	15
AMK-CHL	1
AMK-CTX	1
AMK-CRO	1
AMK-AMP	3
AMK-GEN	3
AMK-SXT	1
AMK-TET	11
AMP-CHL	1
AMP-PIP	5
AMP-SXT	1
AMP-TET	3
CHL-TET	1
CTX-AMP	1
GEN-AMP	1
GEN-CAZ	1
SXT-TET	9
AMK-GEN-AMP	4
AMK-AMP-TET	2
AMK-CTX-AMP	1
AMK-CTX-TET	2
AMK-GEN-TET	2
AMK-SXT-TET	2
AMP-PIP-AMC	1
AMP-PIP-SAM	8
AMP-PIP-SXT	2
AMP-PIP-TET	13
AMP-SAM-TET	2
AMP-SXT-TET	3
CFZ-AMP-AMC	1
CFZ-AMP-PIP	1
FEP-AMP-PIP	1
GEN-AMP-SXT	1
GEN-SXT-TET	1
SXT-CHL-TET	1
AMP-PIP-CHL-TET	4
AMK-AMP-SXT-TET	1
AMK-CTX-AMP-TET	1
AMK-SXT-CHL-TET	1
AMK-GEN-AMP-SXT	1
AMK-GEN-CTX-AMP	1

AMK-GEN-MEN-AMP	1
CFZ-AMP-PIP-CHL	1
CFZ-AMP-PIP-SAM	1
CFZ-AMP-PIP-TET	1
CFZ-CTX-AMP-PIP	1
AMP-PIP-SXT-TET	10
AMP-SAM-CST-TET	1
AMP-SAM-SXT-TET	2
CAZ-CTX-CRO-AMP	1
CFZ-FEP-AMP-PIP	1
CIP-LVX-MXF-TET	1
GEN-AMP-PIP-SXT	1
GEN-AMP-PIP-TET	5
GEN-AMP-SXT-TET	4
SXT-CIP-MXF-TET	1
AMK-AMP-SXT-CHL-TET	3
AMK-GEN-AMP-SXT-TET	6
AMK-CRO-AMP-SXT-CHL	1
AMK-CTX-AMP-CHL-TET	1
AMK-GEN-CTX-CRO-TET	1
CFZ-AMP-PIP-AMC-SAM	1
CFZ-AMP-PIP-CHL-TET	1
CFZ-AMP-PIP-SXT-TET	2
CFZ-CTX-FEP-AMP-PIP	7
AMP-PIP-CIP-LVX-MXF	1
AMP-PIP-SAM-CHL-TET	2
AMP-PIP-SAM-CST-TET	1
AMP-PIP-SAM-SXT-TET	1
AMP-PIP-SXT-CHL-TET	10
AMP-SAM-SXT-CHL-TET	2
CHL-CIP-LVX-MXF-TET	1
CTX-AMP-SXT-CHL-TET	1
GEN-AMP-PIP-CHL-TET	1
GEN-AMP-PIP-SAM-SXT	1
GEN-AMP-PIP-SAM-TET	2
GEN-AMP-PIP-SXT-TET	2
GEN-CTX-CRO-AMP-SXT	1
IPM-AMC-SAM-SXT-TET	1
SXT-CIP-LVX-MXF-TET	1
IPM-CFZ-AMP-PIP-AMC	1
GEN-CFZ-AMP-PIP-SXT-TET	1
GEN-CFZ-CTX-FEP-AMP-PIP	3
GEN-CTX-CRO-AMP-SXT-TET	1
AMK-GEN-AMP-PIP-SAM-TET	1
AMK-CTX-AMP-SXT-CHL-TET	1
AMK-GEN-CAZ-CTX-CRO-AMP	1
AMK-GEN-CTX-AMP-SXT-TET	2
AMK-GEN-CTX-CRO-AMP-SXT	2
AMP-PIP-CIP-LVX-MXF-TET	1
CFZ-AMP-PIP-AMC-CHL-TET	1
CFZ-AMP-PIP-SAM-CHL-TET	1

AMP-PIP-SAM-SXT-CHL-TET	1
AMP-CHL-CIP-LVX-MXF-TET	1
AMP-SXT-CHL-CIP-LVX-MXF	1
CFZ-CTX-FEP-ATM-AMP-PIP	5
GEN-AMP-PIP-SXT-CHL-TET	4
CTX-CRO-AMP-SXT-CHL-TET	1
AMK-GEN-MEN-CTX-CRO-AMP	1
AMK-GEN-CAZ-CTX-CRO-AMP-SXT	1
AMK-GEN-CAZ-CTX-CRO-AMP-TET	2
AMK-CAZ-CTX-CRO-AMP-CHL-TET	1
AMK-CAZ-CTX-CRO-AMP-SXT-TET	2
AMK-GEN-CTX-AMP-SXT-CHL-TET	2
AMK-GEN-CTX-CRO-AMP-SXT-TET	1
AMP-PIP-SXT-CIP-LVX-MXF-TET	14
CFZ-CTX-AMP-PIP-SXT-CHL-TET	3
CFZ-CTX-FEP-AMP-PIP-SXT-TET	1
CFZ-CTX-FEP-AMP-PIP-SAM-CHL	1
AMP-SXT-CHL-CIP-LVX-MXF-TET	2
CFZ-CTX-FEP-ATM-AMP-PIP-TET	1
CFZ-FEP-AMP-PIP-SAM-SXT-TET	1
CFZ-FEP-ATM-AMP-PIP-SAM-TET	1
CFZ-CTX-FEP-ATM-AMP-PIP-CST	1
GEN-AMP-PIP-CIP-LVX-MXF-TET	1
IPM-CFZ-AMP-PIP-AMC-SXT-TET	1
GEN-CFZ-CTX-FEP-AMP-PIP-TET	1
GEN-CFZ-CTX-AMP-PIP-SAM-TET	1
GEN-CFZ-CTX-FEP-AMP-PIP-SAM	4
GEN-AMP-PIP-SXT-CHL-MXF-TET	1
GEN-CAZ-CTX-CRO-AMP-SXT-TET	1
GEN-CTX-CRO-AMP-SXT-CHL-TET	1
GEN-MEN-CAZ-AMP-SXT-CHL-TET	1
AMK-GEN-AMP-PIP-SAM-SXT-CHL-TET	5
AMK-GEN-CAZ-CTX-CRO-AMP-SXT-TET	5
AMK-CAZ-CTX-CRO-AMP-SXT-CHL-TET	5
AMK-GEN-CTX-CRO-AMP-SXT-CHL-TET	1
AMP-PIP-SXT-CHL-CIP-LVX-MXF-TET	1
AMP-SAM-SXT-CHL-CIP-LVX-MXF-TET	1
CFZ-CTX-FEP-AMP-PIP-SXT-CHL-TET	3
CFZ-CTX-FEP-ATM-AMP-PIP-SAM-CHL	1
CFZ-CTX-FEP-ATM-AMP-PIP-SAM-TET	1
CFZ-CTX-FEP-ATM-AMP-PIP-SAM-TZP	1
GEN-CFZ-CTX-AMP-PIP-SAM-CHL-TET	1
GEN-CFZ-CTX-AMP-PIP-SAM-MXF-TET	3
GEN-CFZ-CTX-AMP-PIP-SXT-CHL-TET	1
GEN-CFZ-CTX-FEP-AMP-PIP-CHL-TET	1
GEN-AMP-PIP-SXT-CIP-LVX-MFX-TET	1
GEN-CFZ-CTX-FEP-AMP-PIP-SXT-TET	2
GEN-CFZ-CTX-FEP-ATM-AMP-PIP-TET	3
GEN-CAZ-CTX-CRO-AMP-SXT-CHL-TET	2
AMK-GEN-CFZ-AMP-PIP-SAM-SXT-CHL-TET	1
AMK-GEN-AMP-PIP-SXT-CIP-LVX-MXF-TET	1

AMK-GEN-AMP-PIP-AMC-SAM-SXT-CHL-TET	1
AMK-GEN-CAZ-CTX-CRO-AMP-SXT-CHL-TET	12
CFZ-CAZ-CTX-FEP-ATM-AMP-PIP-CHL-TET	1
CFZ-CTX-FEP-AMP-PIP-AMC-SAM-CHL-TET	1
CFZ-CTX-FEP-ATM-AMP-PIP-CIP-LVX-MXF	2
CFZ-CTX-FEP-ATM-AMP-PIP-SAM-CHL-TET	1
CFZ-CTX-FEP-ATM-AMP-PIP-SXT-CHL-TET	4
GEN-AMP-PIP-SAM-SXT-CHL-CIP-MXF-TET	1
GEN-AMP-PIP-SAM-SXT-CIP-LVX-MXF-TET	3
GEN-AMP-PIP-SXT-CHL-CIP-LVX-MXF-TET	4
GEN-CFZ-CTX-FEP-AMP-PIP-SAM-MXF-TET	1
GEN-CFZ-CTX-FEP-AMP-PIP-SXT-CHL-TET	2
GEN-CFZ-CTX-FEP-ATM-AMP-PIP-SAM-TET	1
GEN-CFZ-FEP-AMP-PIP-SAM-SXT-CHL-TET	1
AMK-GEN-CFZ-CTX-FEP-AMP-PIP-SAM-SXT-TET	4
AMK-GEN-AMP-PIP-SAM-SXT-CIP-LVX-MXF-TET	2
AMK-GEN-CFZ-CTX-FEP-ATM-AMP-PIP-SXT-TET	2
CFZ-CAZ-CTX-FEP-ATM-AMP-PIP-SAM-CHL-TET	1
CFZ-CAZ-AMP-AMC-SAM-SXT-CHL-CIP-LVX-MXF	1
AMK-GEN-MEN-CAZ-CTX-CRO-AMP-SXT-CHL-TET	1
CFZ-CTX-FEP-ATM-AMP-PIP-CIP-LVX-MXF-TET	2
CFZ-CTX-FEP-ATM-AMP-PIP-SAM-CST-SXT-TET	1
CFZ-CTX-FEP-ATM-AMP-PIP-SAM-CIP-LVX-MXF	1
CFZ-CTX-FEP-ATM-AMP-PIP-CST-CIP-LVX-MXF	1
CFZ-CTX-FEP-ATM-AMP-PIP-SAM-SXT-CHL-TET	5
GEN-CFZ-CTX-FEP-AMP-PIP-SAM-CIP-MXF-TET	1
GEN-CFZ-AMP-PIP-SXT-CHL-CIP-LVX-MXF-TET	1
GEN-CFZ-AMP-PIP-SAM-SXT-CIP-LVX-MXF-TET	1
GEN-CFZ-CTX-AMP-PIP-CHL-CIP-LVX-MXF-TET	1
GEN-CFZ-CTX-FEP-ATM-AMP-PIP-SAM-CHL-TET	1
GEN-CFZ-CTX-FEP-ATM-AMP-PIP-SAM-MXF-TET	1
GEN-CFZ-CTX-FEP-ATM-AMP-PIP-SXT-CHL-TET	2
CFZ-CTX-FEP-ATM-AMP-PIP-SXT-CIP-LVX-MXF-TET	2
AMK-GEN-CFZ-CTX-FEP-AMP-PIP-SAM-CHL-CIP-TET	1
AMK-GEN-CFZ-CTX-FEP-ATM-AMP-PIP-SAM-SXT-TET	2
AMK-GEN-CFZ-CTX-ATM-AMP-PIP-CIP-LVX-MXF-TET	1
GEN-CFZ-AMP-PIP-SAM-SXT-CHL-CIP-LVX-MXF-TET	1
GEN-CFZ-CTX-AMP-PIP-SXT-CHL-CIP-LVX-MXF-TET	2
GEN-CFZ-CTX-FEP-AMP-PIP-CST-SXT-CIP-LVX-MXF	1
GEN-CFZ-CTX-FEP-ATM-AMP-PIP-CIP-LVX-MXF-TET	8
GEN-CFZ-CTX-FEP-ATM-AMP-PIP-SAM-CIP-MXF-TET	1
GEN-CFZ-CTX-FEP-ATM-AMP-PIP-SXT-CHL-MXF-TET	1
GEN-CFZ-FEP-AMP-PIP-SAM-CHL-CIP-LVX-MXF-TET	1
GEN-CFZ-FEP-AMP-PIP-SXT-CHL-CIP-LVX-MXF-TET	1
AMK-GEN-CFZ-CTX-FEP-AMP-PIP-SXT-CIP-LVX-MXF-TET	1
AMK-GEN-CFZ-CTX-FEP-ATM-AMP-PIP-SAM-SXT-CHL-TET	1
AMK-GEN-CFZ-AMP-PIP-AMC-SAM-SXT-CIP-LVX-MXF-TET	1
GEN-CFZ-AMP-PIP-AMC-SAM-SXT-CHL-CIP-LVX-MXF-TET	1
CFZ-CTX-FEP-ATM-AMP-PIP-SAM-CHL-CIP-LVX-MXF-TET	1
CFZ-CTX-FEP-ATM-AMP-PIP-SXT-CHL-CIP-LVX-MXF-TET	3
GEN-CFZ-CTX-FEP-AMP-PIP-SAM-CHL-CIP-LVX-MXF-TET	1

GEN-CFZ-CTX-FEP-AMP-PIP-SXT-CHL-CIP-LVX-MXF-TET	9
GEN-CFZ-CTX-FEP-ATM-AMP-PIP-SAM-SXT-CHL-MXF-TET	1
CFZ-CTX-FEP-ATM-AMP-PIP-CST-SXT-CHL-CIP-LVX-MXF-TET	1
CFZ-CTX-FEP-ATM-AMP-PIP-SAM-SXT-CHL-CIP-LVX-MXF-TET	1
AMK-GEN-CFZ-CTX-FEP-ATM-AMP-PIP-SXT-CIP-LVX-MXF-TET	1
GEN-CFZ-CTX-FEP-AMP-PIP-SAM-SXT-CHL-CIP-LVX-MXF-TET	2
GEN-CFZ-CAZ-AMP-PIP-AMC-SAM-SXT-CHL-CIP-LVX-MXF-TET	1
GEN-CFZ-CAZ-CTX-FEP-ATM-AMP-PIP-CHL-CIP-LVX-MXF-TET	1
GEN-CFZ-CTX-FEP-ATM-AMP-PIP-SAM-SXT-CIP-LVX-MXF-TET	1
GEN-CFZ-CTX-FEP-ATM-AMP-PIP-SXT-CHL-CIP-LVX-MXF-TET	10
AMK-GEN-CFZ-CTX-FEP-AMP-PIP-SAM-CST-SXT-CHL-CIP-LVX-MXF	1
AMK-GEN-CFZ-CTX-FEP-AMP-PIP-SAM-SXT-CHL-CIP-LVX-MXF-TET	1
AMK-GEN-CFZ-CAZ-CTX-FEP-ATM-AMP-PIP-SAM-CIP-LVX-MXF-TET	1
AMK-GEN-CFZ-CTX-FEP-ATM-AMP-PIP-SAM-SXT-CIP-LVX-MXF-TET	1
AMK-GEN-CFZ-CTX-FEP-ATM-AMP-PIP-SXT-CHL-CIP-LVX-MXF-TET	4
CFZ-CAZ-CTX-FEP-ATM-AMP-PIP-SAM-SXT-CHL-CIP-LVX-MXF-TET	1
GEN-CFZ-CAZ-CTX-FEP-ATM-AMP-PIP-SAM-SXT-CIP-LVX-MXF-TET	1
GEN-CFZ-CTX-FEP-ATM-AMP-PIP-SAM-SXT-CHL-CIP-LVX-MXF-TET	5
GEN-CFZ-FEP-ATM-AMP-PIP-SAM-CST-SXT-CHL-CIP-LVX-MXF-TET	1
IPM-MEN-CFZ-CAZ-CTX-FEP-AMP-PIP-AMC-SAM-TZP-SXT-CHL-TET	1
AMK-GEN-CFZ-CAZ-CTX-FEP-ATM-AMP-PIP-SAM-SXT-CIP-LVX-MXF-TET	1
AMK-GEN-CFZ-CTX-FEP-ATM-AMP-PIP-SAM-SXT-CHL-CIP-LVX-MXF-TET	1
AMK-GEN-CFZ-CTX-ATM-AMP-PIP-AMC-SAM-SXT-CHL-CIP-LVX-MXF-TET	1
GEN-CFZ-CAZ-CTX-FEP-ATM-AMP-PIP-SAM-SXT-CHL-CIP-LVX-MXF-TET	1
GEN-CFZ-CTX-FEP-ATM-AMP-PIP-SAM-CST-SXT-CHL-CIP-LVX-MXF-TET	1
AMK-GEN-CFZ-CAZ-CTX-FEP-ATM-AMP-PIP-AMC-SAM-SXT-CIP-LVX-MXF-TET	1
AMK-GEN-CFZ-CAZ-CTX-FEP-ATM-AMP-PIP-SAM-SXT-CHL-CIP-LVX-MXF-TET	2
GEN-CFZ-CAZ-CTX-FEP-ATM-AMP-PIP-AMC-SAM-TZP-SXT-CIP-LVX-MXF-TET	1
GEN-IPM-CFZ-CAZ-CTX-FEP-ATM-AMP-PIP-AMC-SAM-TZP-SXT-CHL-CIP-LVX-MXF-TET	1
GEN-IPM-MEN-CFZ-CAZ-CTX-FEP-ATM-AMP-PIP-AMC-SAM-TZP-SXT-CIP-LVX-MXF-TET	1
AMK-GEN-IPM-MEN-CFZ-CAZ-CTX-FEP-AMP-PIP-AMC-SAM-TZP-CST-SXT-CHL-CIP-LVX-MXF-TET	1

Table S2. Percentages of antimicrobial resistance in *E. coli* of different origins and years.

**Antibiotic**	**Pet dogs**			**Working dogs**		**Pet cats**
	**2012-2013 (*n*=78)**	**2015 (*n*=57)**	**2021 (*n*=157)**	**2012-2013 (*n*=51)**	**2021 (*n*=140)**	**2021 (*n*=71)**
Amikacin	74.36%	17.54%	2.55%	78.43%	11.43%	14.08%
Gentamicin	60.26%	59.65%	28.03%	31.37%	40.00%	35.21%
Imipenem	-[a]	0.00%	1.91%	-[a]	2.86%	0.00%
Meropenem	5.13%	0.00%	0.00%	0.00%	2.14%	0.00%
Cefazolin	-[a]	50.88%	41.40%	-[a]	37.86%	63.38%
Ceftazidime	44.87%	7.02%	3.82%	1.96%	3.57%	4.23%
Cefotaxime	56.41%	45.61%	34.39%	27.45%	30.00%	57.75%
Cefepime	-[a]	43.86%	34.39%	-[a]	25.71%	57.75%
Aztreonam	-[a]	19.30%	22.93%	-[a]	16.43%	45.07%
Ampicillin	83.33%	75.44%	74.52%	41.18%	75.71%	83.10%
Piperacillin	-[a]	71.93%	68.79%	-[a]	68.57%	81.69%
Amoxicillin-Clavulanate	-[a]	3.51%	3.18%	-[a]	6.43%	5.63%
Ampicillin-Sulbactam	-[a]	31.58%	21.66%	-[a]	32.14%	33.80%

Piperacillin-Tazobactam	- [a]	0.00%	0.64%	-	2.14%	2.82%
Colistin	0%	5.26%	3.82%	0%	0.71%	1.41%
Trimethoprim-Sulfamethoxazole	67.95%	49.12%	50.32%	25.49%	50.71%	36.62%
Chloramphenicol	38.46%	28.07%	33.76%	11.76%	42.86%	23.94%
Ciprofloxacin	- [a]	49.12%	32.48%	- [a]	20.71%	33.80%
Levofloxacin	- [a]	47.37%	31.85%	- [a]	18.57%	33.80%
Moxifloxacin	- [a]	47.37%	33.12%	- [a]	25.00%	35.21%
Tetracycline	71.79%	59.65%	65.61%	54.90%	80.00%	60.56%
Ceftriaxone	56.41%	- [a]	- [a]	3.92%	- [a]	- [a]

[a] The antibiotic resistance was not tested.

Table S3. Drug resistance pattern of *E. coli*.

Resistance pattern	Pet dogs			Working dogs		Pet cats
	**2012-2013 (*n*=78)**	**2015 (*n*=57)**	**2021 (*n*=157)**	**2012-2013 (*n*=51)**	**2021 (*n*=140)**	**2021 (*n*=71)**
0R	5.13%	17.54%	16.56%	7.84%	16.43%	11.27%
1R	3.85%	1.75%	7.64%	17.65%	4.29%	5.63%
2R	14.10%	7.02%	13.38%	33.33%	6.43%	11.27%
3R	7.69%	8.77%	7.64%	19.61%	12.86%	11.27%
4R	19.23%	10.53%	17.83%	7.84%	10.71%	9.86%
5R	19.23%	14.04%	3.18%	7.84%	7.14%	2.82%
6R	28.21%	8.77%	5.73%	5.88%	17.14%	7.04%
7R	2.56%	3.51%	6.37%	0.00%	10.00%	21.13%
8R	0.00%	12.28%	8.92%	0.00%	7.14%	8.45%
9R	-	14.04%	8.92%	-	4.29%	4.23%
10R	-	1.75%	2.55%	-	2.14%	4.23%
11R	-	0.00%	0.64%	-	0.71%	2.82%
12R	-	0.00%	0.64%	-	0.71%	0.00%

Table S4. Percentage of antimicrobial resistance in ESBL-*E. coli* of different origins and years.

Antibiotic	Pet dogs			Working dogs	Pet cats
	**2012-2013 (*n*=41)**	**2015 (*n*=27)**	**2021 (*n*=60)**	**2021 (*n*=48)**	**2021 (*n*=43)**
Amikacin	14.63%	29.63%	6.67%	12.50%	23.26%
Gentamicin	70.73%	85.19%	58.33%	72.92%	53.49%
Imipenem	0.00%	0.00%	1.67%	6.25%	0.00%
Meropenem	0.00%	0.00%	0.00%	6.25%	0.00%
Cefazolin	100.00%	100.00%	100.00%	100.00%	100.00%
Ceftazidime	19.51%	14.81%	8.33%	10.42%	6.98%
Cefotaxime	100.00%	96.30%	90.00%	87.50%	95.35%
Cefepime	95.12%	92.59%	90.00%	72.92%	95.35%
Aztreonam	73.17%	40.74%	58.33%	47.92%	74.42%
Ampicillin	100.00%	100.00%	100.00%	100.00%	100.00%
Piperacillin	100.00%	100.00%	100.00%	100.00%	100.00%
Amoxicillin-Clavulanate	4.88%	3.70%	1.67%	10.42%	4.65%
Ampicillin-Sulbactam	41.46%	48.15%	38.33%	52.08%	37.21%
Piperacillin-Tazobactam	2.44%	0.00%	1.67%	6.25%	4.65%
Colistin	0.00%	11.11%	6.67%	2.08%	2.33%

Trimethoprim-Sulfamethoxazole	90.24%	51.85%	76.67%	56.25%	44.19%
Chloramphenicol	58.54%	44.44%	71.67%	52.08%	32.56%
Ciprofloxacin	80.49%	66.67%	53.33%	33.33%	55.81%
Levofloxacin	78.05%	62.96%	53.33%	29.17%	55.81%
Moxifloxacin	78.05%	62.96%	55.00%	43.75%	58.14%
Tetracycline	82.93%	62.96%	83.33%	97.92%	76.74%

Table S5. Drug resistance pattern of ESBL-*E. coli*.

		Pet dogs		Working dogs	Pet cats
	**2012-2013**	**2015**	**2021**	**2021**	**2021**
3R[a]	0.00%	3.70%	6.67%	0.00%	11.63%
4R[a]	4.88%	11.11%	3.33%	4.17%	4.65%
5R[a]	4.88%	18.52%	5.00%	6.25%	4.65%
6R[a]	12.20%	3.70%	13.33%	22.92%	11.63%
7R[a]	17.07%	3.70%	15.00%	25.00%	34.88%
8R[a]	24.39%	25.93%	23.33%	18.75%	13.95%
9R[a]	14.63%	29.63%	23.33%	12.50%	6.98%
10R[a]	21.95%	3.70%	6.67%	6.25%	6.98%
11R[a]	0.00%	0.00%	1.67%	2.08%	4.65%
12R[a]	0.00%	0.00%	1.67%	2.08%	0.00%
≥ 7R[a]	78.05%	62.96%	71.67%	66.67%	67.44%

[a] The antibiotics categories.

Table S6. Detection rates of drug resistance genes.

	**Genes**	**AG-type (AMK&GEN) (*n*=34)**	**A-type (AMK) (*n*=34)**	**G-type (GEN) (*n*=111)**	**Drug sensitive type (*n*=74)**
aminoglycosides	*armA*	8.82% (3/34)	8.82% (3/34)	0%	0%
	rmtB	91.18% (31/34)	91.18% (31/34)	0.90% (1/111)	0%
	aac (3)-II	55.88% (19/34)	55.88% (19/34)	72.97% (81/111)	1.35% (1/74)
	aac (6′)-Ib	29.41% (10/34)	29.41% (10/34)	15.32% (17/111)	2.70% (2/74)
trimethoprim-sulfamethoxazole	**Genes**	**Drug resistant type (*n*=143)**	**Drug sensitive type (*n*=76)**		
	sul1	48.95% (70/143)	7.89% (6/76)	-	
	sul2	79.02% (113/143)	25% (19/76)		
	sul3	19.58% (28/143)	1.32% (1/76)		
phenicols	**Genes**	**Drug resistant type (*n*=118)**	**Drug sensitive type (*n*=82)**	**Intermediately sensitive (*n*=19)**	
	folR	80.51% (95/118)	0%	0%	-
	cmlA	31.36% (37/118)	10.98% (9/82)	52.63% (10/19)	
fluoroquinolones	**Genes**	**Resistant to any antibiotic (CIP/LVX/MXF) (*n*=130)**	**Sensitive or intermediately sensitive (*n*=89)**		
	qnrA	0%	0%	-	
	qnrB	1.54% (2/130)	1.12% (1/89)		
	qnrC	0%	0%		
	qnrS	16.15% (21/130)	47.19% (42/89)		
	aac (6′)-Ib-cr	9.23% (12/130)	5.62% (5/89)		

	Genes	Drug resistant type (*n*=181)	Drug sensitive type (*n*=38)	
tetracyclines	*tet (A)*	85.08% (154/181)	2.63% (1/38)	-
	tet (B)	21.55% (39/181)	0%	
	tet (M)	3.31% (6/181)	0%	
	tet (X1-X5)	0%	0%	

Table S7. Phylogenetic grouping of *E. coli* in 2021.

phylogenetic grouping	All	pet dogs	pet cats	working dogs
A	37.77%	33.76%	5.63%	58.57%
B1	30.16%	30.57%	35.21%	27.14%
B2	16.03%	17.20%	42.25%	1.43%
C	2.99%	0.64%	4.23%	5.00%
D	1.09%	2.55%	0.00%	0.00%
E	4.89%	5.73%	2.82%	5.00%
F	6.79%	8.92%	9.86%	2.86%
E.clades	0.27%	0.64%	0.00%	0.00%
B2+D	17.12%	19.75%	42.25%	1.43%
A+C	40.76%	34.39%	9.86%	63.57%

Table S8. Phylogenetic grouping of ESBL-*E. coli.*

Phylogenetic grouping	2012–2013 Pet dogs	2015 Pet dogs	2021 Pet dogs	2021 Pet cats	2021 Working dogs
A	29.27%	0.00%	26.67%	2.33%	47.92%
B1	26.83%	29.63%	30.00%	41.86%	33.33%
B2	2.44%	25.93%	18.33%	30.23%	2.08%
C	4.88%	25.93%	1.67%	6.98%	6.25%
D	4.88%	0.00%	3.33%	0.00%	0.00%
E	9.76%	11.11%	6.67%	2.33%	6.25%
F	19.51%	7.41%	13.33%	16.28%	4.17%
E.clades	2.44%	0.00%	0.00%	0.00%	0.00%
B2+D	7.32%	25.93%	21.67%	30.23%	2.08%
A+C	34.15%	25.93%	28.33%	9.30%	54.17%

Table S9. Collection Information of *E. coli.*

Sampling date	Isolate ID	Age	Gender	Disease	Location	Animal
2012-2013	s4a	<1	Unknown	Diarrheic	Jilin Veterinary Research Institute	Pet dog
	s7a	<1	Male	Diarrheic		
	s9a	<1	Male	Diarrheic		
	s10	<1	Female	Diarrheic		
	s17a	<1	Female	Others		
	s21a	<1	Male	Others		
	z3a	1–5	Female	Diarrheic	Zhou Ping Pet Hospital	Pet dog
	z4a	>10	Male	Others		
	z5e	1–5	Male	Others		

z6a	<1	Female	Diar-rheic		
z7a	1–5	Female	Others		
z8a	<1	Female	Diar-rheic		
z9a	<1	Female	Others		
z10a	<1	Male	Diar-rheic		
z11a	<1	Female	Diar-rheic		
z12a	<1	Female	Diar-rheic		
z13a	<1	Male	Diar-rheic		
z14a	<1	Female	Diar-rheic		
z16a	<1	Female	Diar-rheic		
z17a	<1	Male	Diar-rheic		
z18a	<1	Male	Diar-rheic		
z22a	6–10	Male	Others		
z23-2a	6–10	Female	Others		
z23-3a	6–10	Female	Others		
z23-4a	1–5	Male	Others		
z24a	<1	Male	Diar-rheic		
z25a	<1	Female	Others		
z28a	<1	Male	Others		
z30a	<1	Female	Diar-rheic		
z34a	1–5	Male	Health		
z35a	1–5	Male	Health		
z36a	<1	Female	Others		
z40a	<2	Female	Diar-rheic		
z41a	1–5	Male	Others		
z42a	<1	Male	Diar-rheic		
z43a	<1	Male	Diar-rheic		
z45a	1–5	Male	Others		
z46a	<1	Male	Others		
z47a	<1	Male	Others		
z48a	<1	Male	Others		
1123a	Un-known	Un-known	Un-known	Chuncheng Pet Hospital	Pet dog
1126-1a	Un-known	Un-known	Un-known		

1126-3a	Un-known	Un-known	Un-known
1128a	Un-known	Un-known	Un-known
1129-2a	Un-known	Un-known	Un-known
1130-1a	Un-known	Un-known	Un-known
1130-2b	Un-known	Un-known	Un-known
1130d	Un-known	Un-known	Un-known
1141a	Un-known	Un-known	Un-known
1158c	Un-known	Un-known	Un-known
1160a	Un-known	Un-known	Un-known
1202-1a	Un-known	Un-known	Un-known
1202-2a	Un-known	Un-known	Un-known
1203a	Un-known	Un-known	Un-known
1207-1a	Un-known	Un-known	Diar-rheic
1207-2b	Un-known	Un-known	Un-known
1208-2a	Un-known	Un-known	Un-known
1209-1a	Un-known	Un-known	Un-known
1211-2a	<1	Un-known	Diar-rheic
1212-2a	Un-known	Un-known	Diar-rheic
1215-1a	Un-known	Un-known	Un-known
1215-2a	Un-known	Un-known	Un-known
1217-2a	Un-known	Un-known	Un-known
1217-3c	Un-known	Un-known	Un-known
1219a	Un-known	Un-known	Un-known
1221-2a	Un-known	Un-known	Un-known
1226-1a	Un-known	Un-known	Un-known

	1228-2a	Un-known	Un-known	Un-known		
	1228c	Un-known	Un-known	Un-known		
	1230-1a	Un-known	Un-known	Un-known		
	130107-1a	Un-known	Un-known	Un-known		
	130107-2d	Un-known	Un-known	Un-known		
	C2a	Un-known	Un-known	Un-known		
	C4a	Un-known	Un-known	Un-known		
	c6a	Un-known	Un-known	Un-known		
	c7a	Un-known	Un-known	Un-known		
	c8a	Un-known	Un-known	Un-known		
	c9a	Un-known	Un-known	Un-known		
2015	528-2	>10	Male	Others	Zhou Ping Pet Hospital	Pet dog
	528-4	6-10	Male	Others		
	528-11	<1	Female	Others		
	528-12	<1	Male	Others		
	528-14	6-10	Male	Others		
	528-15	6-10	Female	Un-known		
	528-16	6-10	Female	Others		
	528-17	<1	Male	Others		
	528-19	<1	Female	Others		
	528-21	1–5	Female	Others		
	528-22	1–5	Female	Others		
	528-23	1–5	Male	Un-known		
	604-24	<1	Female	Others		
	529-41	1–5	Male	Diar-rheic		
	601-42	<1	Male	Others		
	601-43	1-5	Female	Diar-rheic		
	601-44	6–10	Female	Others		
	529-45	<1	Male	Others		
	603-46	6–10	Male	Others		
	601-48-2	<1	Female	Others		
	603-53	<1	Male	Others		
	601-61	<1	Male	Others		
	601-62	Un-known	Un-known	Others		

601-64	1-5	Female	Diar-rheic
603-66-2	<1	Female	Un-known
601-67	6–10	Female	Others
601-68	<1	Female	Others
603-71	1–5	Female	Diar-rheic
601-78	Un-known	Female	Others
529-79	<1	Female	Un-known
604-80	<1	Male	Un-known
601-81	<1	Female	Un-known
601-82	1-5	Male	Un-known
601-83	<1	Male	Others
603-86	<2	Male	Others
603-88	1–5	Female	Un-known
603-90	Un-known	Male	Others
604-93	1–5	Male	Un-known
604-96	Un-known	Female	Diar-rheic
604-100	<1	Male	Diar-rheic
604-101	Un-known	Male	Others
604-103	Un-known	Male	Un-known
603-109	>10	Male	Others
604-111	Un-known	Female	Un-known
604-112	<1	Female	Un-known
603-113	<1	Female	Un-known
529-a2	6–10	Male	Others
529-a3	>10	Male	Others
529-a4	1–5	Female	Others
529-a5	1–5	Female	Health
529-a6	1–5	Female	Diar-rheic
603-a9	1–5	Female	Others
603-c1	1–5	Female	Others
604-C6	<1	Male	Others
603-CB2	Adult	Male	Others

	604-CB3	Adult	Female	Others		
	604-CB4	Adult	Female	Others		
2021	HP121	>10	Male	Diarrheic	Zhou Ping Pet Hospital	Pet dog
	HP123	1-5	Male	Health		
	HP124	1-5	Female	Others		
	HP125	>10	Female	Health		
	HP126	1–5	Male	Diarrheic		
	HP129	1–5	Female	Others		
	HP131	<1	Male	Unknown		
	HP132	<1	Male	Unknown		
	HP133	<1	Male	Unknown		
	HP134	<1	Female	Unknown		
	HP135	<1	Female	Unknown		
	HP136	<1	Female	Unknown		
	HP137	<1	Female	Unknown		
	HP138	1–5	Female	Unknown		
	HP140	>10	Female	Unknown		
	HP142	1–5	Unknown	Unknown		
	HP143	1–5	Unknown	Unknown		
	HP144	1–5	Male	Others		
	HP145	Unknown	Unknown	Unknown		
	HP146	1–5	Female	Unknown		
	HP147	1–5	Male	Health		
	HP148	1–5	Male	Others		
	HP152	1–5	Male	Unknown		
	HP155	1–5	Male	Unknown		
	HP156	1–5	Female	Unknown		
	HP157	1–5	Female	Unknown		
	HP158	1–5	Male	Unknown		
	HP160	1–5	Male	Unknown		

HP161	1–5	Female	Un-known
HP162	1–5	Male	Un-known
HP164	1–5	Male	Un-known
HP169	1–5	Male	Un-known
HP174	<1	Female	Un-known
HP175	<1	Female	Un-known
HP176	1–5	Female	Un-known
HP179	1–5	Male	Un-known
HP181	1–5	Male	Un-known
HP182	6–10	Female	Un-known
HP192	1–5	Male	Un-known
HP194	Un-known	Un-known	Un-known
HP196	6–10	Female	Un-known
HP197	<1	Male	Un-known
HP198	1–5	Male	Others
HP199	1–5	Female	Health
HP200	1–5	Male	Others
HP201	<1	Male	Diar-rheic
HP203	1–5	Male	Health
HP204	1–5	Female	Health
HP206	<1	Male	Diar-rheic
HP207	Un-known	Un-known	Un-known
HP208	<1	Male	Health
HP209	<1	Male	Health
HP210	<1	Male	Diar-rheic
HP211	<1	Male	Diar-rheic
HP213	<1	Male	Health
HP214	<1	Male	Diar-rheic
HP219	6–10	Female	Others
HP221	1–5	Female	Diar-rheic

HP222	Un-known	Female	Others
HP224	<1	Un-known	Diar-rheic
HP225	<1	Female	Diar-rheic
HP226	1–5	Female	Others
HP230	>10	Female	Others
HP232	6–10	Female	Others
HP233	6–10	Female	Others
HP235	6–10	Female	Others
HP236	1–5	Male	Others
HP237	6–10	Un-known	Others
HP238	1–5	Male	Others
HP239	<1	Male	Others
HP241	1–5	Male	Diar-rheic
HP242	6–10	Un-known	Health
HP244	1–5	Male	Others
HP245	1–5	Male	Others
HP246	6–10	Female	Others
HP248	1–5	Un-known	Others
HP249	6–10	Male	Others
HP252	1–5	Un-known	Others
HP254	<1	Male	Others
HP256	1–5	Un-known	Others
HP259	1–5	Un-known	Others
HP260	1–5	Un-known	Others
HP261	1–5	Un-known	Health
HP262	6–10	Female	Others
HP263	1–5	Un-known	Others
HP264	<1	Un-known	Others
HP265	<1	Un-known	Others
HP266	<1	Un-known	Others
HP267	1–5	Un-known	Health
HP268	1–5	Female	Un-known

HP270	<1	Unknown	Others		
HP271	<1	Female	Diarrheic		
HP272	1–5	Female	Others		
HP273	<1	Male	Diarrheic		
HP276	<1	Female	Others		
HP277	>10	Female	Others		
HP278	>10	Female	Others		
HP279	1-5	Female	Health		
HP280	6-10	Female	Health		
HP282	<1	Unknown	Health		
HP283	<1	Unknown	Health		
HP284	1–5	Male	Health		
HP286	1–5	Unknown	Health		
HP287	1–5	Unknown	Health		
HP288	1–5	Unknown	Health		
HP289	<1	Unknown	Health		
HP290	<1	Unknown	Health		
HP292	<1	Unknown	Health		
HP293	<1	Unknown	Diarrheic		
HP296	<1	Female	Health		
HP297	<1	Male	Health		
HP300	1–5	Unknown	Others		
HP302	<1	Unknown	Diarrheic		
HP305	<1	Unknown	Others		
HP306	Unknown	Unknown	Unknown		
HC1-1dc	Unknown	Unknown	Unknown		
HC1-3dc	Unknown	Unknown	Unknown		
HC1-5dc	Unknown	Unknown	Unknown	Chong Yisheng, Central Hospital	Pet dog
HC1-6dc	Unknown	Unknown	Unknown		
HC1-8dc	Unknown	Unknown	Unknown		

HC1-14dc	Un-known	Un-known	Un-known		
HC1-15dc	Un-known	Un-known	Un-known		
HC1-16dc	Un-known	Un-known	Un-known		
HC1-18dc	Un-known	Un-known	Un-known		
HC1-20dc	Un-known	Un-known	Un-known		
HC1-22dc	Un-known	Un-known	Un-known		
HC1-24dc	Un-known	Un-known	Un-known		
HC1-26dc	Un-known	Un-known	Un-known		
HC1-27dc	Un-known	Un-known	Un-known		
HC1-28dc	Un-known	Un-known	Un-known		
HC1-32dc	Un-known	Un-known	Un-known		
HC1-37dc	Un-known	Un-known	Un-known		
HC1-38dc	Un-known	Un-known	Un-known		
HC1-39dc	Un-known	Un-known	Un-known		
HC1-40dc	Un-known	Un-known	Un-known		
HC1-41dc	Un-known	Un-known	Un-known		
HC1-43dc	Un-known	Un-known	Un-known		
HC1-46dc	Un-known	Un-known	Un-known		
HC1-48dc	Un-known	Un-known	Un-known		
HC1-49dc	Un-known	Un-known	Un-known		
HC1-51dc	Un-known	Un-known	Un-known		
HC1-52dc	Un-known	Un-known	Un-known		
HC1-55dc	Un-known	Un-known	Un-known		
HC1-57dc	Un-known	Un-known	Un-known		
HC2-2dc	Un-known	Un-known	Un-known	Chong Yisheng, Yangpu Street	Pet dog

	HC2-9dc	Unknown	Unknown	Unknown		
	HC2-31dc	Unknown	Unknown	Unknown		
	HC2-33dc	Unknown	Unknown	Unknown		
	HC2-50-1dc	Unknown	Unknown	Unknown		
	HC2-51dc	Unknown	Unknown	Unknown		
	HC3-4dc	Unknown	Unknown	Unknown	Animal Hospital of Jilin Agricultural University	Pet dog
	HC3-10dc	Unknown	Unknown	Unknown		
	HC4-3dc	Unknown	Unknown	Unknown	Dr. xie	Pet dog
	HC4-7dc	Unknown	Unknown	Unknown		
	HC4-10dc	Unknown	Unknown	Unknown		
	HC4-13dc	Unknown	Unknown	Unknown		
	HC4-14dc	Unknown	Unknown	Unknown		
2021	HP1	6–10	Male	Health	Zhou Ping Pet Hospital	Pet cat
	HP2	6–10	Male	Others		
	HP3	1–5	Male	Others		
	HP7	<1	Male	Unknown		
	HP10	<1	Male	Health		
	HP11	1–5	Male	Health		
	HP12	1–5	Male	Diarrheic		
	HP13	1–5	Female	Health		
	HP14	1–5	Female	Health		
	HP15	1–5	Male	Diarrheic		
	HP16	1–5	Male	Others		
	HP17	<1	Female	Diarrheic		
	HP20	<1	Female	Diarrheic		
	HP23	<1	Female	Health		
	HP24	<1	Male	Health		
	HP25	Unknown	Unknown	Unknown		
	HP27	1–5	Male	Others		
	HP28	<1	Female	Diarrheic		
	HP31	Unknown	Unknown	Diarrheic		

HP34	<1	Un-known	Un-known
HP39	1–5	Male	Others
HP40	<1	Male	Others
HP42	>10	Female	Others
HP44	6–10	Un-known	Un-known
HP45	1–5	Male	Others
HP46	<1	Male	Diar-rheic
HP53	<1	Un-known	Health
HP54	<1	Male	Health
HP55	<1	Male	Health
HP56	<1	Male	Health
HP60	1–5	Male	Others
HP61	1–5	Female	Others
HP62	1–5	Male	Others
HP64	<1	Male	Diar-rheic
HP65	1–5	Male	Health
HP67	<1	Male	Others
HP69	1–5	Female	Health
HP72	<1	Un-known	Others
HP73	1–5	Un-known	Others
HP74	<1	Female	Health
HP75	<1	Un-known	Diar-rheic
HP76	1–5	Un-known	Others
HP77	<1	Un-known	Others
HP78	<1	Un-known	Others
HP79	1–5	Un-known	Health
HP80	1–5	Un-known	Health
HP86	<1	Male	Health
HP87	1–5	Un-known	Others
HP88	1–5	Male	Others
HP90	<1	Un-known	Diar-rheic
HP91	1–5	Un-known	Health
HP93	<1	Un-known	Diar-rheic

HP94	1–5	Un-known	Others
HP95	<1	Male	Health
HP97	1–5	Female	Health
HP98	1–5	Male	Health
HP99	1–5	Male	Health
HP100	1–5	Un-known	Others
HP101	1–5	Un-known	Others
HP102	>10	Un-known	Others
HP107	1–5	Male	Health
HP110	1–5	Male	Others
HP111	1–5	Male	Others
HP112	<1	Female	Diar-rheic
HP113	1–	Un-known	Health
HP114	<1	Male	Diar-rheic
HP115	<1	Un-known	Others
HP116	Un-known	Un-known	Un-known
HP117	Un-known	Un-known	Un-known
HP118	Un-known	Un-known	Un-known
HP120	Un-known	Un-known	Un-known

Table S10. PCR primers and conditions.

Target	Primer	Sequence (5′–3′)	Fragment size	Reference
E.coli 16S rDNA	F	TGTGGGAACGGCGAGTCGGAATAC	1467	[54]
	R	GGGCGCAGGGGATGAAACTCAAC		
chuA	chuA.1b	ATGGTACCGGACGAACCAAC	288	[57]
	chuA.2	TGCCGCCAGTACCAAAGACA		
yjaA	yjaA.1b	CAAACGTGAAGTGTCAGGAG	211	
	yjaA.2b	AATGCGTTCCTCAACCTGTG		
TspE4C2	TspE4C2.1b	CACTATTCGTAAGGTCATCC	152	
	TspE4C2.2b	AGTTTATCGCTGCGGGTCGC		
arpA	AceK.f	AACGCTATTCGCCAGCTTGC	400	
	ArpA1.r	TCTCCCCATACCGTACGCTA		
bla_{CTX-M}	CTX-MU1	ATGTGCAGYACCAGTAARGT	544	[58]
	CTX-MU2	TGGGTRAARTARGTSACCAGA		
bla_{SHV}	SHV-F	ATTTGTCGCTTCTTTACTCGC	1018	
	SHV-R	TTTATGGCGTTACCTTTGACC		

*bla*TEM	TEM-F<br>TEM-R	ATGAGTATTCAACATTTCCGT<br>TTACCAATGCTTAATCAGTGA	861	[59]
*bla*OXA-1	O-for<br>O-rev	GGCACCAGATTCAACTTTCAAG<br>GACCCCAAGTTTCCTGTAAGTG	564	[60]
tet (A)	tet(A)-F<br>tet(A)-R	GTAATTCTGAGCACTGTCGC<br>CTGCCTGGACAACATTGCTT	956	[61]
tet (B)	tet(B)-F<br>tet(B)-R	CTCAGTATTCCAAGCCTTTG<br>ACTCCCCTGAGCTTGAGGGG	414	
tet (M)	tet(M)-F<br>tet(M)-R	GTGGACAAAGGTACAACGAG<br>CGGTAAAGTTCGTCACACAC	406	[62]
tet (X1)	tet(X1) -F<br>tet(X1) -R	CGAAAAATGTTGCTTGGCAGCTT<br>AGTTGTTGAACGAATTAACTCC	486	[63]
tet (X2)	tet(X2) -F<br>tet(X2) -R	CGGGATGTCCAAGGTAAGAAAA<br>TGACAACGTCGTATGAATCAA	343	
tet (X3)	tet(X3) -F<br>tet(X3) -R	GACACTTGATCTGCACAGGGATT<br>CCCTACAAAAGATGATGTCAAAC	685	
tet (X4)	tet(X4) -F<br>tet(X4) -R	CTGATTCGTGTGACATCATCTTTTG<br>GTTAAATTTCCCATTGGTCAGATTA	204	
tet (X5)	tet(X5) -F<br>tet(X5) -R	GGTATCAACATTTCAATGCTTG<br>CGATTCGTCCTGCGTATCTTTTG	265	
sul1	sul1-F<br>sul1-R	CACCGGAAACATCGCTGCA<br>AAGTTCCGCCGCAAGGCT	158	[64]
sul2	sul2-F<br>sul2-R	CTCCGATGGAGGCCGGTAT<br>GGGAATGCCATCTGCCTTGA	190	
sul3	sul3-F<br>sul3-R	CCCATACCCGGATCAAGAATAA<br>CAGCGAATTGGTGCAGCTACTA	143	
floR	floR-F<br>floR-R	GGCTTTCGTCATTGCGTCTC<br>ATCGGTAGGATGAAGGTGAGGA	650	[47]
cmlA	cmlA-F<br>cmlA-F	TGCCAGCAGTGCCGTTTAT<br>CACCGCCCAAGCAGAAGTA	900	
qnrA	qnrA-F<br>qnrA-R	ATTTCTCACGCCAGGATTTG<br>GATCGGCAAAGGTTAGGTCA	516	[65]
qnrB	qnrB-F<br>qnrB-R	GATCGTGAAAGCCAGAAAGG<br>ACGATGCCTGGTAGTTGTCC	469	
qnrS	qnrS-F<br>qnrS-R	ACGACATTCGTCAACTGCAA<br>TAAATTGGCACCCTGTAGGC	417	
qnrC	qnrC-F<br>qnrC-R	GGGTTGTACATTTATTGAATCG<br>CACCTACCCATTTATTTTCA	307	[66]
aac (6′)-Ib-cr	aac (6′)-Ib-cr-F<br>aac (6′)-Ib-cr-R	ATATGCG-<br>GATCCAAAAACAAAGTTAGGCATCACAAAG<br>ATATGCGAATTCCTTCAGTTCCTTCAAA-<br>TAATGGAGA	810	[67]
rmtB	rmtB-F<br>rmtB-R	ACATCAACGATGCCCTCAC<br>AAGTTCTGTTCCGATGGTC	725	[68]
armA	armA-F<br>armA-R	ATGGATAAGAATGATGTTGTTAAG<br>TTATTTCTGAAATCCACTAGTAATTA	774	[69]
aac(3)- II	aac(3)- II -F<br>aac(3)- II -R	ACTGTGATGGGATACGCGTC<br>CTCCGTCAGCGTTTCAGCTA	237	
aac (6′)-Ib	aac (6′)-Ib-F<br>aac (6′)-Ib-R	ATGACTGAGCATGACCTTGC<br>TTAGGCATCACTGCGTGTTC	519	
*bla*IMP	IMP-F	GGAATAGAGTGGCTTAAYTCTC	232	[70]

	IMP-R	GGTTTAAYAAAACAACCACC		
*bla*KPC	KPC-F	ATGTCACTGTATCGCCGTCT	893	[71]
	KPC-R	TTTTCAGAGCCTTACTGCCC		
*bla*NDM	NDM-F	TCGCCCCATATTTTTGCTAC	1080	[72]
	NDM-R	CTGGGTCGAGGTCAGGATAG		
mcr-1	F	AGTCCGTTTGTTCTTGTGGC	320	
	R	AGATCCTTGGTCTCGGCTTG		
mcr-2	F	CAAGTGTGTTGGTCGCAGTT	715	
	R	TCTAGCCCGACAAGCATACC		
mcr-3	F	AAATAAAAATTGTTCCGCTTATG	929	[73]
	R	AATGGAGATCCCCGTTTTT		
mcr-4	F	TCACTTTCATCACTGCGTTG	1116	
	R	TTGGTCCATGACTACCAATG		
mcr-5	F	ATGCGGTTGTCTGCATTTATC	1644	
	R	TCATTGTGGTTGTCCTTTTCTG		

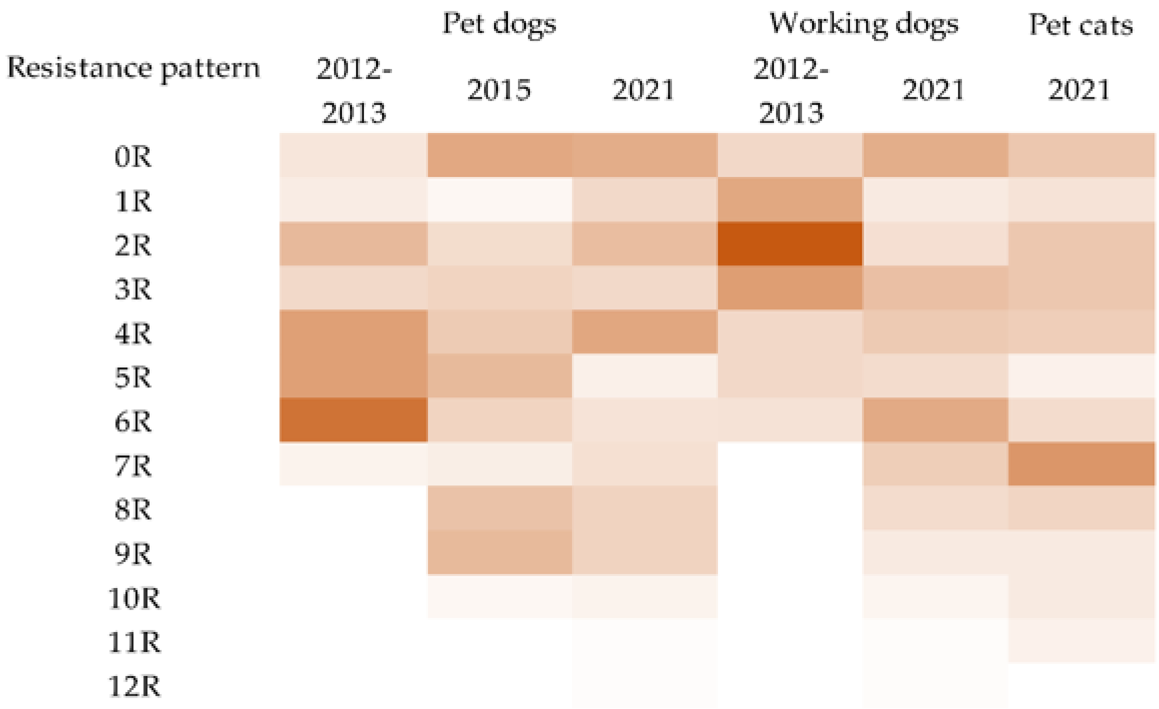

Figure S1. Drug resistance pattern of E. coli.
